# [Retracted] Paeoniflorin inhibits nucleus pulposus cell apoptosis by regulating the expression of Bcl-2 family proteins and caspase-9 in a rabbit model of intervertebral disc degeneration

**DOI:** 10.3892/etm.2025.12907

**Published:** 2025-06-16

**Authors:** Lijun Shi, Honglin Teng, Minyu Zhu, Chi Li, Kelun Huang, Bi Chen, Yusen Dai, Jing Wang

Exp Ther Med 10:257–262, 2015; DOI: 10.3892/etm.2015.2501

Following the publication of the above paper, it was drawn to the Editor’s attention by a concerned reader that, regarding the flow cytometric plots shown in Fig. 3A on p. 260, three of the data panels appeared to show similar groupings of dots, which would not have been anticipated if these experiments had been performed discretely under different experimental conditions, suggesting a fundamental flaw either in the way in which these experiments were performed, or in how the results were outputted. Moreover, some of these flow cytometric data were strikingly similar to data that had already appeared in an another article written by different authors at different research institutes in *Yonsei Medical Journal*.

The Editor of *Experimental and Therapeutic Medicine* has decided that this paper should be retracted from the Journal on account of a lack of confidence in the presented data, and also given that these data had apparently been published previously. The authors were asked for an explanation to account for these concerns, but the Editorial Office did not receive a reply. The Editor apologizes to the readership for any inconvenience caused.

